# Fostering a comprehensive understanding of neurodegenerative biomarkers through collaborations: Integration of findings from a randomized controlled trial and postmortem datasets

**DOI:** 10.1002/alz70856_107212

**Published:** 2026-01-08

**Authors:** Hye Won Chai, Dariusz Pytel, Abigail Stephan, Jennifer Belk, Michelle Ackerman, Laura Davis, Feng Ding, Steven L Carroll, Lesley A. Ross

**Affiliations:** ^1^ Clemson University, Institute for Engaged Aging, Seneca, SC, USA; ^2^ Medical University of South Carolina, Charleston, SC, USA; ^3^ Clemson University, Clemson, SC, USA

## Abstract

**Background:**

Neurodegenerative biomarkers, including Amyloid‐β40, 42, Neurofilament light chain (NfL), Glial fibrillary acidic protein (GFAP), and *p*‐Tau 217, are considered promising hallmarks of Alzheimer's Disease and Related Dementias (AD/ADRD). These biomarkers can be collected antemortem or postmortem, with each providing distinct insights into the role of neurodegenerative biomarkers in AD/ADRD. Biomarkers collected from living individuals provide an understanding of treatment effects and biological responses, while postmortem data offers an understanding of the disease's end‐stage manifestation. Integrating these different sources of data is crucial in enhancing a comprehensive knowledge regarding the biological mechanisms of cognitive decline and AD/ADRD. Therefore, the aims of this study are to introduce a case of collaboration between two institutions with access to two distinct datasets and present integrative results from the findings of each dataset.

**Method:**

Antemortem data comes from the Elucidating the Necessary Active Components of Training (ENACT) data, a randomized controlled trial designed to test the transfer effects of cognitive training. ENACT has collected blood samples from a subsample of its participants (*n* = 60) during baseline and posttest. The samples are currently stored at Clemson University and will be shipped to the Medical University of South Carolina (MUSC) for the analyses of the neurodegenerative biomarkers on MUSC's Quanterix HD‐X system. Aβ40, 42, NfL and GFAP will be analyzed using the NEUROLOGY 4‐PLEX assay and *p*‐Tau 217 will be analyzed using ALZpath *p*‐Tau 217. For the postmortem data from a different source, MUSC has completed HD‐X analysis on postmortem blood and cerebrospinal fluid (CSF) specimen for Amyloid‐β 40,42, GFAP, and NfL.

**Result:**

From ENACT and postmortem datasets, we hypothesize that lower peripheral levels of Amyloid‐β1‐42, higher Amyloid‐β1‐40, lower amyloid‐β 42/40 ratio, higher NfL, and higher *p*‐Tau 217 will each relate to poorer cognition or greater progression of neurodegenerative diseases. From ENACT data specifically, we also expect to identify the mechanistic role of neurodegenerative biomarkers in cognitive training transfer effects.

**Conclusion:**

Findings from this study will provide an example of how collaborations between two institutions with distinct datasets can foster a comprehensive understanding of neurodegenerative biomarkers as they relate to cognitive decline and AD/ADRD pathology.

